# The complex anatomy of the bronchial arteries: a meta-analysis with potential implications for thoracic surgery and hemoptysis treatment

**DOI:** 10.1038/s41598-024-81935-5

**Published:** 2024-12-28

**Authors:** Patryk Ostrowski, Michał Bonczar, Kinga Glądys, Maria Klimeczek-Chrapusta, Agata Musiał, Aleksandra Matuszyk, Krzysztof Balawender, Jerzy Walocha, Mateusz Koziej, Eduard Clarke, Michał Polguj, Anna Smędra, Andrzej Żytkowski, Grzegorz Wysiadecki

**Affiliations:** 1 Department of Anatomy, Jagiellonian University Medical College, Kraków, Poland; 2 Youthoria, Youth Research Organization, Kraków, Poland; 3 Department of Normal and Clinical Anatomy, Institute of Medical Sciences, Medical College of Rzeszow University, Rzeszów, Poland; 4 Laboratory of Neuroanatomy, Department of Normal and Clinical Anatomy, Chair of Anatomy and Histology, Medical University of Lodz, ul. Żeligowskiego 7/9, 90-752 Łódź, Poland; 5 Department of Normal and Clinical Anatomy, Chair of Anatomy and Histology, Medical University of Lodz, Łódź, Poland; 6 Chair and Department of Forensic Medicine, Medical Faculty, Medical University of Lodz, Łódź, Poland; 7 Department of Anatomy, Faculty of Medicine, University of Social Sciences in Lodz, Łódź, Poland; 8 Norbert Barlicki Memorial Teaching Hospital No. 1 of the Medical University of Lodz, Łódź, Poland

**Keywords:** Anatomical variations, Bronchial artery, Bronchial branches, Hemoptysis, Bronchial artery embolization, Anatomy, Medical research

## Abstract

The present meta-analysis aimed to provide the most detailed and comprehensive anatomical description of bronchial arteries (BAs) using data available in the literature. Adequate knowledge of the normal anatomy and morphological variations of BAs can be clinically significant; for example, this approach can prevent potential risks while undertaking bronchial artery embolization (BAE) procedures and, ultimately, lead to better patient outcomes. Major medical databases such as PubMed, Scopus, Embase, Web of Science, Google Scholar, and the Cochrane Library were searched. The overall search process was conducted in three main stages. The number of BAs varied from one to six, and 16 arterial patterns were observed. The most common variation was in one right BA and one left BA, with a pooled prevalence of 19.54% (95% CI 6.69–36.44%). The pooled prevalence of BAs originating separately from the aorta was 41.42% (95% CI 37.42–45.48%). The number and location of BAs are highly inconsistent. However, the most prevalent pattern involved two BAs: one in the right BA and one in the left BA. Although BAs most frequently originate from the descending aorta, the cooccurrence of at least one ectopic BA is relatively high. The results of our meta-analysis can serve as a source of comprehensive information for thoracic surgeons and physicians performing endovascular procedures, especially BAE, a treatment for life-threatening hemoptysis.

## Introduction

The lungs have a dual arterial supply that consists of the pulmonary and bronchial arterial systems. Pulmonary circulation shunts deoxygenated blood from the heart to the lungs (where the blood is resaturated with oxygen) and returns the oxygenated blood to the left atrium. The bronchial arteries (BAs) supply the bronchial tree, lymph nodes, large blood vessels, esophagus, and pleura^1–3^. The bronchial circulation is a low-capacity, high-pressure system that increases blood flow in various disease processes, including inflammatory disease, tumors, and congenital heart disease, possibly resulting in hypertrophy of BAs. Consequently, in many disorders, BAs are a frequent source of hemoptysis^4^.

The anatomy of the cardiovascular system is subject to significant variability^5–8^. Similarly, the origin and distribution patterns of BAs vary immensely. Typically, BAs arise, mainly separately, from the descending aorta between the fifth and sixth thoracic vertebrae. The BAs that originate outside the Th5-Th6 vertebral levels of the thoracic aorta or from the other aortic branches are considered ectopic and are also defined as aberrant or anomalous^9^. The prevalence of an anomalous origin of BAs is said to vary between 8 and 35% ^4^. The most frequently reported origins of ectopic BAs in the literature have been the aortic arch, descending aorta, and subclavian arteries^4,10–12^.

The bronchial arteries are a frequent source of hemoptysis^4^. Massive hemoptysis, defined as the expectoration of blood or blood-tinged sputum from the lower respiratory tract, is a relatively common and life-threatening condition^13–15^. The in-hospital mortality rate for hemoptysis patients is estimated to be approximately 9.4% ^16^. Bronchial artery embolization (BAE) is a noninvasive treatment for patients with hemoptysis and is characterized by a high success rate; the immediate clinical success of BAE ranges between 70 and 99% ^17^. However, the anomalous origin of BAs, presented in various anatomical and angiographic studies, increases the difficulty of the procedure and increases the number of recurrences. BAE has been proven effective at controlling life-threatening hemoptysis, but precise knowledge of the location of the bleeding artery is essential for this procedure. Therefore, the present meta-analysis aimed to provide the most detailed description of the anatomy of BAs using data available in the literature. Adequate knowledge of the normal anatomy and variations in BAs can decrease the number of potential risks while undertaking BAE procedures and, ultimately, lead to better patient outcomes.

## Materials and methods

### Search strategy

For the sake of this meta-analysis, a systematic complex search was conducted in which all articles regarding, or at least mentioning, the anatomy of the BAs were examined. Major medical databases, such as PubMed, Scopus, Embase, Web of Science, Google Scholar, and the Cochrane Library, were searched. The overall search process was conducted in 3 stages. (1) In the first step, all the above-mentioned medical databases were searched using the following search terms: [bronchial AND (artery OR arteries OR vessel OR vessels)]. No date, language, article type, or text availability conditions were applied. (2) Furthermore, the mentioned databases were searched through once again using another set of search phrases: (a) (bronchial artery[Title/Abstract]) AND (anatomy [Title/Abstract]); (b) (bronchial artery[Title/Abstract]) AND (morphology [Title/Abstract]); (c) (bronchial artery[Title/Abstract]) AND (topography [Title/Abstract]); (d) (bronchial artery[Title/Abstract]) AND (variation [Title/Abstract]); (e) (bronchial artery[Title/Abstract]) AND (pattern [Title/Abstract]); (f) (bronchial artery[Title/Abstract]) AND (surgery [Title/Abstract]); (g) (bronchial artery[Title/Abstract]) AND (transplantation [Title/Abstract]); (h) (bronchial artery[Title/Abstract]) AND (embolization [Title/Abstract]); (i) (bronchial [Title/Abstract]) AND (branch [Title/Abstract]); (j) (pulmonary [Title/Abstract]) AND (arterial supply [Title/Abstract]); (k) (pulmonary [Title/Abstract]) AND (blood supply [Title/Abstract]). Additionally, each phrase has been checked for the dependence of the results on grammatical variations of a given phrase. (3) Later, an additional manual search was also performed for all references from the initial submitted studies. The Preferred Reporting Items for Systematic Reviews and Meta-Analyses (PRISMA) guidelines were followed. Additionally, the Critical Appraisal Tool for Anatomical Meta-analysis (CATAM) and Anatomical Quality Assessment Tool (AQUA) were used to provide the highest quality findings^18,19^.

### Eligibility assessment and data extraction

**Fig. 1:**
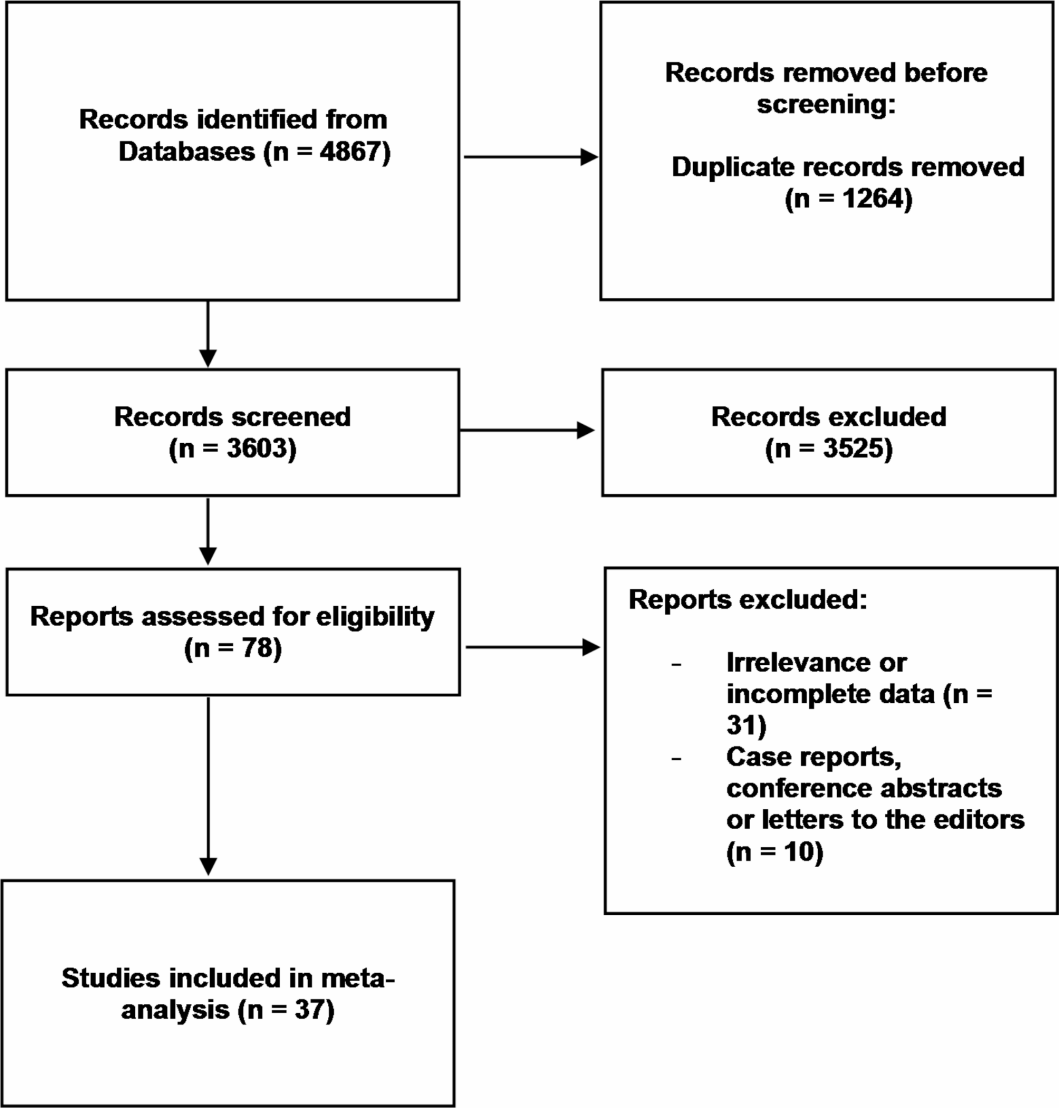
Flow diagram presenting the process of collecting the data included in this meta-analysis.

The following inclusion criteria were established: original articles with extractable data on the anatomy, morphology, topography, and variations of the bronchial arteries. The exclusion criteria included conference reports, case reports, case series, reviews, letters to the editor, papers describing patients with a noticeable pathology that could distort the BA anatomy, and studies with no relevant or incompatible data. Two independent scientists performed a systematic search and initially evaluated 4867 articles. Finally, 37 articles met the required criteria and were used in this meta-analysis^4,9–12,20–51^. The overall process of data collection is presented in Fig. 1. The characteristics of the included studies can be found in Table 1. Two independent researchers extracted the relevant data from the eligible studies.

**Table 1:** Characteristics of studies included in this meta-analysis.

First author	Year	Continent	Country	Method	Number of individuals	AQUA assessment
Choi, WS	2021	Asia	South Korea	MDCT angiography + digital subtraction angiography	600	D1: low
						D2: low
						D3: low
						D4: low
						D5: low
Maeda, T	2021	Asia	Japan	Computed tomography	79	D1: low
						D2: unclear
						D3: low
						D4: low
						D5: low
Le, HY	2020	Asia	Vietnam	Computed tomography	57	D1: low
						D2: low
						D3: low
						D4: low
						D5: low
Michimoto, K	2020	Asia	Japan	Angiography	39	D1: high
						D2: low
						D3: low
						D4: unclear
						D5: low
Befera, N	2019	Europe	Great Britain	Angiography	180	D1: low
						D2: low
						D3: low
						D4: unclear
						D5: unclear
Ying, C	2019	Asia	China	Angiography	108	D1: low
						D2: low
						D3: low
						D4: low
						D5: low
Fei, QL	2018	Asia	China	Cadavers	48	D1: unclear
						D2: unclear
						D3: low
						D4: low
						D5: low
Mori, K	2018	Asia	Japan	Computed Tomography	31	D1: high
						D2: low
						D3: low
						D4: low
						D5: low
Esparza-Hernández, CN	2017	North America	Mexico	Angiography	139	D1: low
						D2: low
						D3: low
						D4: low
						D5: low
Hayasaka, K	2017	Asia	Japan	Cadavers	72	D1: low
						D2: unclear
						D3: low
						D4: low
						D5: low
Kocbek, L	2017	Europe	Slovenia	Cadavers	43	D1: low
						D2: unclear
						D3: unclear
						D4: low
						D5: low
Cuesta, MA	2016	Europe	The Netherlands	Surgery	25	D1: high
						D2: high
						D3: low
						D4: low
						D5: low
Hayasaka, K	2016	Asia	Japan	Cadavers	72	D1: low
						D2: low
						D3: unclear
						D4: low
						D5: low
Yener, O	2015	Asia	Turkey	Computed tomography	208	D1: low
						D2: low
						D3: low
						D4: low
						D5: low
Bhalla, A	2015	Asia	India	Computed tomography	334	D1: low
						D2: unclear
						D3: unclear
						D4: unclear
						D5: high
Kajiyama, Y	2014	Asia	Japan	Surgery	124	D1: low
						D2: high
						D3: high
						D4: high
						D5: low
T. Wada	2012	Asia	Japan	Angiography	64	D1: low
						D2: low
						D3: low
						D4: low
						D5: low
Ziyawudong, J	2012	Asia	Japan	Angiography	100	D1: low
						D2: low
						D3: low
						D4: low
						D5: low
Battal, B	2011	Asia	Turkey	MDCT angiography	163	D1: low
						D2: low
						D3: low
						D4: low
						D5: low
Morita, Y	2010	Asia	Japan	Computed tomography	73	D1: low
						D2: low
						D3: low
						D4: low
						D5: low
Yu, H	2010	Asia	China	Computed tomography	39	D1: low
						D2: low
						D3: low
						D4: low
						D5: low
Kotoulas, C	2010	Europe	Greece	Biopsies	40	D1: low
						D2: low
						D3: low
						D4: unclear
						D5: low
Hartmann, IJC	2007	Europe	France	Angiography	214	D1: low
						D2: low
						D3: low
						D4: low
						D5: low
Yoon, YC	2005	Asia	South Korea	Computed tomography + angiography	22 + 20	D1: high
						D2: low
						D3: low
						D4: low
						D5: unclear
Remy-Jardin, M	2004	Europe	France	MDCT angiography	48	D1: low
						D2: low
						D3: low
						D4: low
						D5: unclear
Kuiper, S	2003	Australia	New Zealand	Computed tomography + cadavers	90 + 22	D1: low
						D2: low
						D3: low
						D4: low
						D5: low
Tanomkiat, W	2003	Asia	Thailand	Angiograms + radiographs	43	D1: low
						D2: unclear
						D3: low
						D4: low
						D5: low
Sancho, C	1998	Europe	Spain	Angiography	25	D1: low
						D2: low
						D3: low
						D4: low
						D5: low
Funami, Y	1996	Asia	Japan	Cadavers	71	D1: low
						D2: low
						D3: low
						D4: low
						D5: low
Carles, J	1995	Europe	France	Cadavers + angiography	40 + 50	D1: low
						D2: low
						D3: low
						D4: low
						D5: low
Kauczor, HU	1994	Europe	Germany	Computed tomography	39 + 20	D1: unclear
						D2: unclear
						D3: low
						D4: low
						D5: low
Schwickert, HC	1994	Europe	Germany	Computed tomography	43	D1: low
						D2: low
						D3: low
						D4: low
						D5: low
Dupont, P	1991	Europe	France	Cadavers	24	D1: unclear
						D2: low
						D3: low
						D4: low
						D5: low
Riquet, M	1991	Europe	France	Cadavers	30	D1: low
						D2: low
						D3: low
						D4: low
						D5: low
Schreinemakers, HHJ	1990	North America	USA	Cadavers	30	D1: low
						D2: low
						D3: low
						D4: low
						D5: low
Liebow, AA	1965	North America	USA	Cadavers	50	D1: low
						D2: low
						D3: low
						D4: low
						D5: low
Cauldwell, EW	1948	Europe	United Kingdom	Cadavers	150	D1: low
						D2: low
						D3: low
						D4: low
						D5: low

Qualitative data, such as year of publication, country, and continent, were collected. Subsequently, quantitative data were gathered in several categories: (1) The prevalence of different arrangements of BAs originating from the descending aorta. The overall number of BAs, the side of the aorta from which each BA originated, and the arrangement of those arteries were considered. (2) The prevalence of different types of BAs concerning their origin. In this category, the prevalence of BAs originating in each part of the aorta and the prevalence of BAs originating directly or via a trunk were taken into account. (3) The prevalence of ectopic BAs concerning patient side and sex. Additionally, the artery from which those ectopic BAs originate has also been evaluated. (4) Morphometrical data regarding BAs. In this category, the BA diameter and length were considered. Any discrepancies between the studies identified by the two people were resolved by contacting the authors of the original studies wherever possible or by consensus with a third person.

### Statistical analysis

To perform this meta-analysis, STATISTICA version 13.1 software (StatSoft, Inc., Tulsa, OK, USA), MetaXL version 5.3 software (EpiGear International Pty Ltd., Wilston, Queensland, Australia), and Comprehensive Meta-analysis version 4.0 software (Biostat, Inc., Englewood, NJ, USA) were used. A random effects model was used. The chi-square test and the I-squared statistic were chosen to assess the heterogeneity among the studies^52–54^. P values and confidence intervals were used to determine the statistical significance of the differences between the studies. A p value lower than 0.05 was considered to indicate statistical significance. The differences were considered to be statistically insignificant when the confidence intervals overlapped. I-squared statistics were interpreted as follows: values of 0–40% were considered “might not be important”, values of 30–60% were considered “might indicate moderate heterogeneity”, values of 50–90% were considered “may indicate substantial heterogeneity”, and values of 75–100% were considered “may indicate substantial heterogeneity”^53^. The results obtained using different methods did not significantly differ (*p* > 0.05). Therefore, an overall analysis could be performed.

## Results

**Fig. 2:**
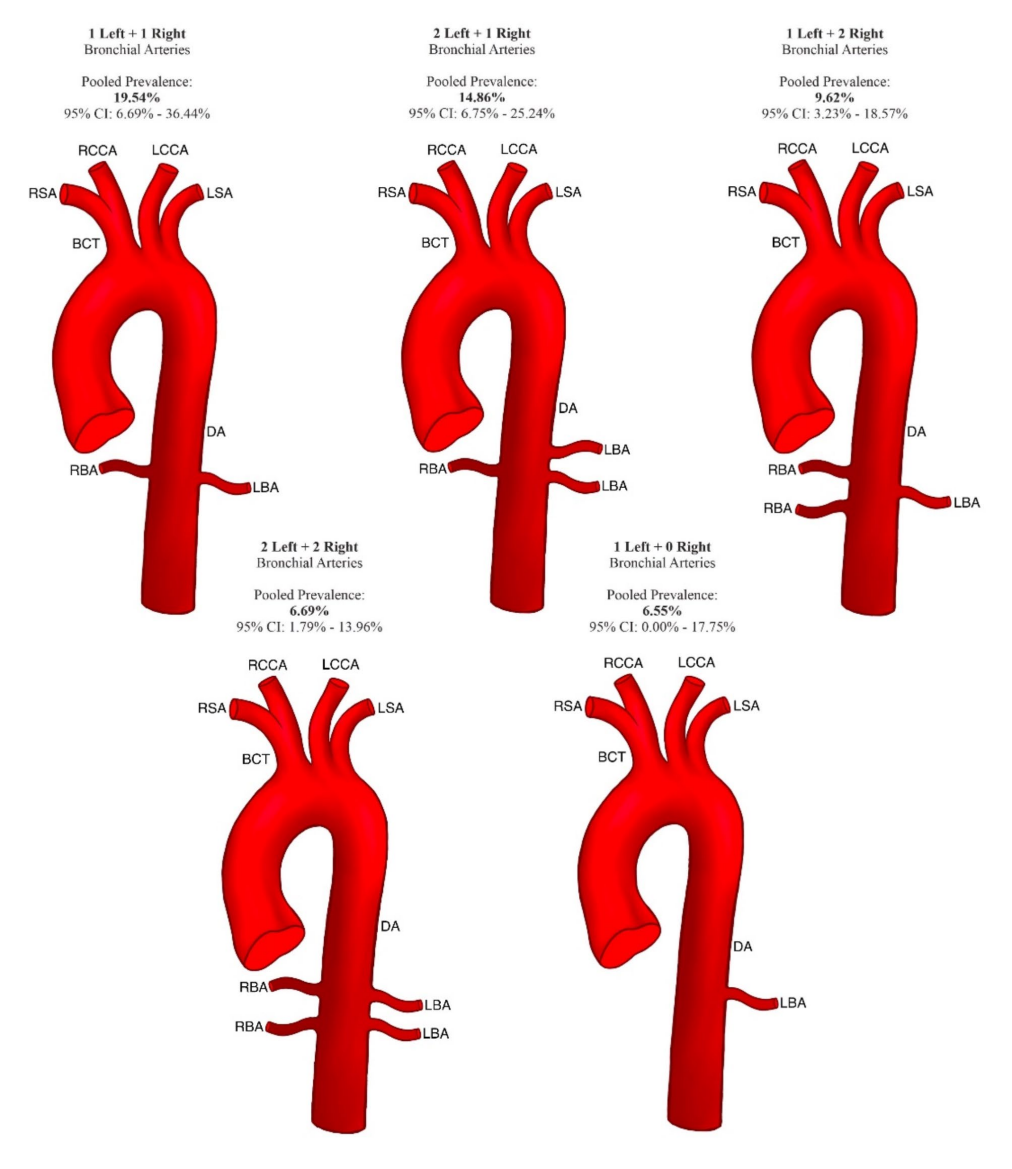
Scheme presenting the five most common variations in the arrangement and number of bronchial arteries established in this meta-analysis. *RBA* right bronchial artery, *LBA* Left bronchial artery, *DA* descending aorta, *BCT* Brachiocephalic trunk, *RSA* right subclavian artery, *RCCA* right common carotid artery, *LCCA* Left common carotid artery, *LSA* Left subclavian artery.

The number of BAs varied from 1 to 6, and the BA distribution was divided into 16 arrangements. The most common variation was one with one right BA and one with one left BA, for a pooled prevalence of 19.54% (95% CI 6.69–36.44%). Subsequently, the pooled prevalence of variation with one right BA and two left BAs was 14.84% (95% CI 6.75–25.24%). The detailed results regarding each arrangement and their pooled prevalence can be found in Table 2. A scheme presenting the five most common variations in arrangement and the number of BAs established in this meta-analysis can be found in Fig. 2.

**Table 2:** Statistical results of this meta-analysis regarding the incidence of each arrangement of the bronchial arteries (BAs). *LCI* lower confidence interval, *HCI* higher confidence interval, *Q* Cochran’s Q.

Number of BAs	Variant	*N*	Pooled prevalence	LCI	HCI	Q	I^2^
1	0 Right + 1 Left	1546	6.55%	0.00%	17.75%	582.76	98.11
1	1 Right + 0 Left	1546	4.90%	0.00%	13.04%	406.31	97.29
2	1 Right + 1 Left	1546	19.54%	6.69%	36.44%	531.20	97.93
2	0 Right + 2 Left	1546	0.51%	0.00%	1.66%	41.81	73.69
3	1 Right + 2 Left	1546	14.86%	6.75%	25.24%	254.01	95.67
3	2 Right + 1 Left	1546	9.62%	3.23%	18.57%	248.22	95.57
4	2 Right + 2 Left	1546	6.69%	1.79%	13.96%	213.12	94.84
4	3 Right + 1 Left	1546	0.75%	0.21%	1.59%	20.62	46.66
4	1 Right + 3 Left	1546	0.67%	0.07%	1.72%	31.95	65.57
5	3 Right + 2 Left	1546	1.40%	0.03%	4.09%	96.34	88.58
5	2 Right + 3 Left	1546	1.13%	0.21%	2.62%	41.64	73.58
5	1 Right + 4 Left	1546	0.20%	0.03%	0.51%	7.07	0.00
5	4 Right + 1 Left	1546	0.18%	0.02%	0.47%	3.70	0.00
6	3 Right + 3 Left	1546	0.15%	0.01%	0.43%	5.32	0.00
6	2 Right + 4 Left	1546	0.21%	0.02%	0.55%	11.94	7.87
6	1 Right + 5 Left	1546	0.15%	0.01%	0.42%	5.69	0.00

**Fig. 3:**
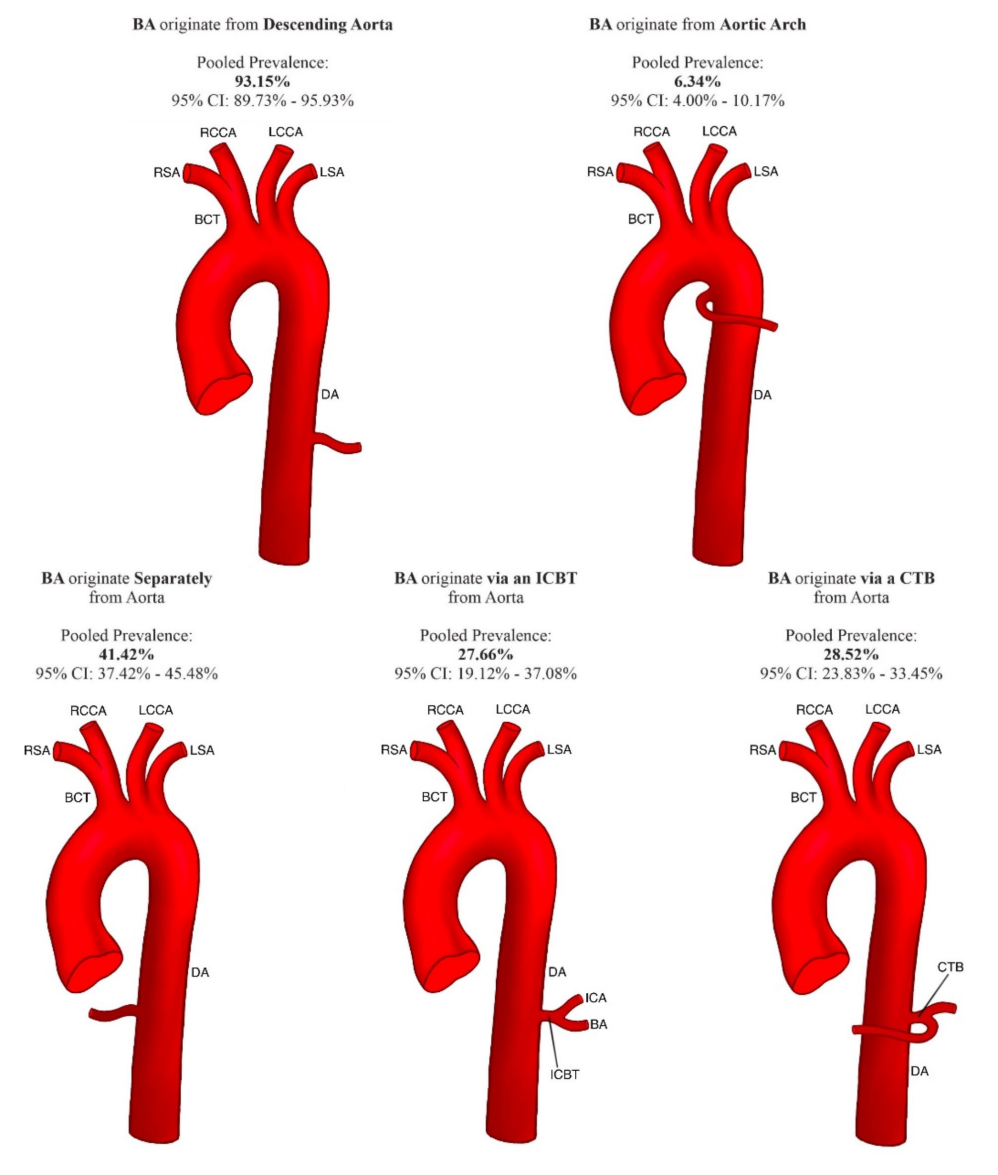
Scheme presenting the variations in the origin of bronchial arteries. *BA* Bronchial artery, *ICBT* intercostal-bronchial trunk, *CTB* common trunk for both bronchial arteries, *ICA* intercostal artery, *DA* descending aorta, *BCT* Brachiocephalic trunk, *RSA* right subclavian artery, *RCCA* right common carotid artery, *LCCA* left common carotid artery, *LSA* left subclavian artery.

The pooled prevalence of BAs originating from the aorta was 92.71% (95% CI 80.91–100.00%). The pooled prevalence of BAs originating separately from the aorta was 41.42% (95% CI 37.42–45.48%). The pooled prevalence of BAs originating from the aorta via the intercostobronchial trunk (ICBT) was set to 27.66% (95% CI 19.12–37.08%). Of the BAs that originated via an ICBT, 95.37% (95% CI 90.65–98.58%) were formed by a right BA. All the above-mentioned results and more detailed results regarding the origin of the BAs can be found in Table 3. A schematic of the variations in the origin of Bas is presented in Fig. 3.

**Table 3:** Statistical results of this meta-analysis regarding the origin of the bronchial arteries (BAs). *LCI* lower confidence interval, *HCI* higher confidence interval, *Q* Cochran’s Q.

Category	*N*	Pooled prevalence	LCI	HCI	Q	I^2^
Pooled prevalence of BA originating from Aorta						
BA originating from Aorta	3273	92.71%	80.91%	100.00%	517.77	99.03
BA **not** originating from Aorta	3273	7.29%	0.00%	19.09%	517.77	99.03
Variations of BA origination from Aorta						
BA originate separately from Aorta	1124	41.42%	37.42%	45.48%	3.72	46.17
BA originate from Aorta via an intercostal-bronchial trunk [ICBT]	1124	27.66%	19.12%	37.08%	21.98	90.90
Prevalence of Right BA forming the ICBT	949	95.37%	90.65%	98.58%	41.23	83.02
Prevalence of Left BA forming the ICBT	949	3.72%	2.45%	5.23%	7.97	12.21
BA originate from Aorta via a common trunk of both bronchial arteries [CTB]	1124	28.52%	23.83%	33.45%	6.22	67.83
BA origin point with respect to Aortas’ part						
BA originate from Descending Aorta	1333	93.15%	89.73%	95.93%	14.19	78.86
BA originate from Aortic Arch	1333	6.34%	4.00%	10.17%	14.19	78.86

The pooled prevalence of at least one ectopic BA was 37.80% (95% CI 25.51–50.91%). The pooled prevalence of ectopic BAs originating from the aortic arch was 70.60% (95% CI 57.61–82.13%). All the above-mentioned results and additional detailed results regarding ectopic BAs can be found in Table 4.

**Table 4:** Statistical results of this meta-analysis regarding ectopic bronchial arteries (BAs). *LCI* lower confidence interval, *HCI* higher confidence interval, *Q* Cochran’s Q.

Category	*N*	Pooled prevalence	LCI	HCI	Q	I^2^
Prevalence of ectopic BAs						
Presence of at least one ectopic BA	1289	37.80%	25.51%	50.91%	106.71	94.38
Presence of at least one ectopic BA in females	149	29.68%	21.36%	38.73%	2.79	28.37
Presence of at least one ectopic BA in males	437	30.85%	26.22%	35.67%	2.37	15.66
Occurrence of the ectopic BAs in respect to patients’ side						
Prevalence of occurrence of ectopic BA on the right side	472	51.43%	41.00%	61.79%	20.71	71.03
Prevalence of occurrence of ectopic BA on the left side	472	49.20%	38.88%	59.56%	20.47	70.69
Origin of the ectopic BAs						
Aortic arch	494	70.60%	57.61%	82.13%	52.92	82.99
Lower descending aorta	494	12.39%	3.69%	24.65%	72.26	87.55
Right subclavian artery	494	4.25%	2.30%	6.73%	10.70	15.86
Left subclavian artery	494	3.87%	2.30%	5.80%	9.12	1.33
Right internal throacic artery	494	2.49%	0.88%	4.80%	12.24	26.45
Left internal thoracic artery	494	2.14%	1.03%	3.62%	4.24	0.00
Left thyrocervical trunk	494	1.78%	0.78%	3.16%	3.29	0.00
Right thyrocervical trunk	494	1.66%	0.15%	4.34%	17.31	48.02
Brachocephalic trunk	494	1.24%	0.00%	3.70%	17.56	48.74
Ascending Aorta	494	0.69%	0.12%	1.66%	3.26	0.00
Left common carotid artery	494	0.52%	0.04%	1.39%	2.74	0.00

The right BA’s mean maximal diameter was 1.64 mm (SE = 0.10). The mean maximal diameter of the left BA was established at 1.48 mm (SE = 0.07). All the above-mentioned results and more detailed results regarding the morphometry of the BAs can be found in Table 5.

**Table 5:** Statistical results of this meta-analysis regarding the mean maximal diameter of the right bronchial artery (RBA) and left bronchial artery (LBA).

Category	Mean (mm)	Standard error	Variance	Lower limit	Upper limit	Z-value	*p*-value
RBA maximal diameter	1.64	0.10	0.01	1.46	1.83	17.30	0.00
LBA maximal diameter	1.48	0.07	0.01	1.34	1.62	20.66	0.00

## Discussion

There is considerable disagreement on which scientists provided the first anatomical description of the BA. It is believed that Leonardo da Vinci discovered bronchial artery circulation. Nevertheless, along with him, many other scientists, such as Galen, Virchow, and Frederick Ruysch, are cited as the ones who share significant contributions in describing the anatomy of the BA^14^. Importantly, the study conducted by Cauldwell et al. in 1948 ^51^ provided the classic description of the branching patterns of BAs. Since then, the results of their study have been used as a reference for many clinical procedures, such as BAE. The classification consisted of four types: type I, two on the left and one on the right, which presented as an ICBT (40% of the patients); type II, one on the left and one ICBT on the right (21% of the patients); type III, two on the left and two on the right (one ICBT and one BA) (20% of the patients); and last, type IV, one on the left and two on the right (one ICBT and one BA) (9.7% of the patients)^51,55^. The most commonly observed distribution pattern was three bronchial arteries—two on the left side and one on the right side^51^. This particular anatomical arrangement is considered one of the reasons why the right main bronchus is more susceptible to ischemia than its left counterpart is. Since the analysis was conducted by Cauldwell et al.^51^, numerous studies regarding the anatomy of BAs have been published in the literature. Yener et al.^11^ conducted a computed tomographic-based study regarding normal anatomy and variations in BAs. In that study, the most common (24%) branching pattern was a combination of 1 right ICBT and 1 left BA, and the second most common (13.46%) was a combination of 2 right (1 ICBT and 1 BA) and 1 left BA. Other studies have presented similar conclusions to those of Cauldwell et al.^51^ regarding the number of BAs, with a greater prevalence on the left side than on the right side^45^.

According to our study, the highest prevalence of branching patterns was associated with the occurrence of two BAs, one located on the left side and one on the right side, corresponding to type II according to Cauldwell’s classification. This contradicts what was thought concerning the ischemia of the bronchi, with the right presumably being more susceptible to it than the left bronchus. Our results show that this is not the case and that the bronchi, in most cases, are supplied by an equal number of BAs.

The origin of the BA has been heavily discussed due to its relevance in endovascular procedures. BAs may originate separately, forming common trunks with each other or other nearby vessels. However, in most cases, BAs originate from the descending aorta. The right BA frequently forms a common trunk with the right intercostal artery, forming the ICBT, which has been reported to be present in up to 58% of cases^24^. However, cases in which left BAs form an ICBT have also been reported in the literature^56^. According to the present meta-analysis, the overall prevalence of a BA originating from an ICBT was 27.66%.

Furthermore, in most cases, the right BA was found to form the ICBT more frequently (95.37%) than the left BA (3.72%). Interestingly, the BAs were found to form common trunks with each other relatively frequently, with an overall prevalence of 28.52%. Choi et al.^10^ conducted a study in which the origin of BAs was analyzed using multidetector computed tomography (OM) in 600 patients. The study showed that the most common origin was the thoracic aorta (87.5%). However, relatively many cases of ectopic origins have been reported (12.5%).

In our study, the prevalence of abnormal or ectopic origins of BA was 37.80%. Furthermore, if the BA had an ectopic origin, the most common origin was from the aortic arch (70.60%), followed by the lower segment of the descending aorta (12.39%) and the right subclavian artery (4.25%). Interestingly, origins from the brachiocephalic trunk and the thyrocervical trunk were also presented in the literature, revealing how superior the location of the origin of the BA may be. In contrast to the origins mentioned above, the BA may be highly inferior in location to its origin. Jiang et al.^57^ reported an aberrant left BA originating from the left gastric artery in a case report concerning a patient with acute massive hemoptysis. In et al.^58^ described this extreme abnormality in a similar case report of a patient with massive hemoptysis. Both of these patients were successfully treated with transarterial embolization.

Although the primary focus of our study is on the anatomical variations of BAs, it is crucial to acknowledge the role of BA hypertrophy and anomalies in clinical settings, particularly their association with hemoptysis and other thoracic pathologies. BA hypertrophy often occurs as a compensatory mechanism in response to chronic pulmonary ischemia, pulmonary embolism, or other pulmonary vascular diseases^14,59^. Under these conditions, systemic blood flow through the BAs increases significantly, from their typical 1% of cardiac output to as much as 18–30% in diseases like chronic thromboembolic disease^59–61^. However, the dilated and hypertrophic BAs, with diameters often exceeding 2 mm, are prone to rupture under systemic pressure, leading to hemoptysis, particularly in patients with underlying chronic inflammatory or infectious diseases^59,62^. BA anomalies are less common but no less significant. Bronchial arteriovenous malformations (BAVMs), a rare congenital or acquired condition, result in abnormal connections between the BAs and pulmonary veins or arteries, forming left-to-right or left-to-left extracardiac shunts^59,63^. These malformations, when acquired, may develop in response to inflammatory lung diseases, trauma, or tumors, and they can lead to life-threatening hemoptysis if left untreated^63,64^.

The embryological development of BAs may explain the occurrence of ectopic origins of these arteries. Adult BAs originate from the process of involution, which occurs in primitive branches originating from the dorsal aorta. These primitive branches supply the pulmonary plexus during embryological development. The persistence of one of these primitive branches may result in an ectopic BA with an abnormally superior origin originating from the brachiocephalic trunk; carotid, subclavian, internal thoracic, or vertebral artery; or thyrocervical trunk, among others^10,65,66^.

Having adequate knowledge about the anatomy of the BA is of enormous importance when performing BAE. Embolization of BAs has been used as a treatment for both benign and malignant causes of hemoptysis^17^. It has been described as highly effective, with a success rate ranging from 68 to 100% ^17,23,67^. A systematic review conducted by Panda et al.^17^ revealed that the most common indications for BAE were the control of hemoptysis due to active tuberculosis and posttuberculosis complications, comprising fibrosis, bronchiectasis, and aspergilloma. However, the indications for BAE are vast and include pathologies such as cystic fibrosis, malignancies, and lung infections, among others. The origin of the BAs plays a vital role in choosing the access site for BAE. Choi et al.^10^ explained that both femoral and radial access were effective when the BAs had a usual or ectopic origin from the descending aorta. However, in patients where the ectopic BA originated from the aortic arch or ascending aorta, the femoral access was straightforward, but radial access was challenging. When an ectopic BA originated from the subclavian artery, carotid artery, or their branches, radial access was more accessible than was femoral access. The present meta-analysis revealed that BAs originate most frequently from the descending aorta (93.15%). However, the prevalence of at least one ectopic BA was relatively high (37.80%). The high probability of an abnormal origin necessitates analyzing the arterial vasculature of the patient prior to a potential embolization procedure. This approach can help surgeons choose the proper access site for the procedure. Furthermore, having proper knowledge of the morphometric properties of the BA, precisely its origin, is highly relevant for choosing suitable catheters prior to the intravascular procedure. The current study showed that, on average, the right and left BAs had diameters of 1.64 mm and 1.48 mm, respectively.

A thorough understanding of the anatomy of BAs holds significant importance in cardiothoracic surgery. Bronchial artery revascularization (BAR) has been proven to increase 5-year survival in patients receiving en bloc double-lung transplants^68^. However, due to the technical complexity of the procedure, BAs are routinely sacrificed and ignored during lung transplants. The occurrence of airway ischemia after lung transplantation remains a significant concern during the perioperative period, as reported prevalence rates range from 2 to 11%, according to recent studies^69–71^. Specifically, patients who are not anatomically suitable for bibronchial anastomosis may necessitate an en bloc double lung transplant, which is associated with a notably high incidence of tracheal complications, reaching up to 40% ^72^. Moreover, accumulating evidence indicates that compromised microvasculature, suboptimal perfusion, and hypoxemia in transplanted lungs play significant roles in the pathogenesis of chronic lung allograft dysfunction (CLAD) and bronchiolitis obliterans syndrome/obliterative bronchiolitis (BOS/OB)^73–75^. The results presented in the current meta-analysis may help to overcome the technical complexity of BAR by providing cardiothoracic surgeons with necessary data concerning the complete anatomy of BAs.

This study has several limitations. This may be burdened by potential bias, as the accuracy of the data collected from various publications limits the results of this meta-analysis. The authors were unable to perform some of the analyses due to an insufficient amount of consistent data. Furthermore, most of the evaluated studies were from Asia and Europe. Therefore, the results of the present study may be burdened with potential bias, as they may reflect the anatomical features of Asian and European people rather than the global population. Furthermore, the study has not been registered in any database (for example: PROSPERO), which might have influenced the potential bias. Despite these limitations, our meta-analysis attempted to estimate BA anatomy based on data from the literature that met the requirements of evidence-based anatomy.

## Conclusion

In conclusion, this is the most precise and up-to-date study on the variable anatomy of the BA. Our results showed that the number and location of BAs are highly inconsistent. However, the most prevalent pattern was two BAs: one in the right BA and one in the left BA. Furthermore, the BA was found to originate most frequently from the descending aorta, but the probability of an individual having at least one ectopic BA is relatively high. The results of the present meta-analysis will be helpful for physicians performing endovascular procedures, especially BAE, as a treatment for life-threatening hemoptysis.

## Data Availability

The authors declare that the data supporting the findings of this study are available within the paper. Should any raw data files be needed in another format they are available from the corresponding author upon reasonable request.
